# Comparison of the Performance Parameters of BioHPP^®^ and Biocetal^®^ Used in the Production of Prosthetic Restorations in Dentistry—Part I: Mechanical Tests: An In Vitro Study

**DOI:** 10.3390/ma18030561

**Published:** 2025-01-26

**Authors:** Robert Kowalski, Wojciech Frąckiewicz, Magdalena Kwiatkowska, Małgorzata Światłowska-Bajzert, Ewa Sobolewska

**Affiliations:** 1 Department of Dental Prosthetics, Faculty of Medicine and Dentistry, Pomeranian Medical University in Szczecin, Av. Powstańców Wlkp. 72, 70-111 Szczecin, Poland; 2 Ra-Dent Stomatologia Protetyka, Bolesława Krzywoustego Street 19/5, 70-252 Szczecin, Poland; 3 Faculty of Mechanical Engineering and Mechatronics, West Pomeranian University of Technology in Szczecin, Av. Piastów 19, 70-310 Szczecin, Poland

**Keywords:** dentistry, Biocetal, BioHPP, hardness, impact strength, abrasive wear, tensile strength, flexural strength

## Abstract

The aim of these in vitro studies was to determine and compare the mechanical and tribological performance of two commercially available thermoplastic materials, namely BioHPP and Biocetal, used in dental prosthetics. In order to perform the comparative tests of both materials, the dog-bone shaped samples were formed by an injection molding process as in standard polymer materials research, wherein Biocetal samples constituted the research group, and BioHPP samples served as a control group. In the presented studies, their mechanical parameters were reported and analyzed: namely, Shore’s hardness, unnotched impact strength, tensile strength, flexural strength, as well as abrasive wear resistance, obtained within appropriate tribological and mechanical tests. The Shapiro–Wilk test, Q–Q plot analysis, Grubbs test and Student’s *t*-test (*p* < 0.05) were used to statistically evaluate the results. The experimental results revealed that BioHPP material is characterized by higher hardness, impact strength, bending strength, and also lower “wet” abrasion wear if compared to Biocetal performance. However, it is subject to higher abrasive wear under “dry” conditions and reveals higher stiffness as well as lower ability to deform, which could affect a patient’s comfort during application. BioHPP, despite being a high-performance polymer material, also has some drawbacks that may affect the poorer long-term use of dentures in people producing less saliva.

## 1. Introduction

The entire stomatognathic system undergoes physiological and pathological changes as the body matures. This results in tooth loss, which impacts the patient’s ability to chew and speak in addition to their smile aesthetics [1]. Dental prostheses that reconstruct complete dental arches as well as individual gaps allow for the restoration of these functions. For the purpose of creating prostheses that are strong, aesthetically pleasing, biocompatible, and comfortable for the patient, researchers are always searching for ever-better materials [2]. Polymethyl methacrylate (PMMA) dentures are the most widely used restorations because they are inexpensive and simple to make. However, thermoplastic dentures, like those made of polyoxymethylene (POM) or polyetheretherketone (PEEK), have superior aesthetics and durability, and they can sometimes take the place of traditional acrylic dentures in clinical settings [3].

The study describes two thermoplastic materials—polyoxymethylene, POM, in dental prosthetics available as Biocetal (ROKO Dental Systems, Częstochowa, Poland) [4] and a new-generation BioHPP material (BredentGmbH, Senden, Germany), which is a semi-crystalline polymer based on polyetheretherketone, PEEK, containing 20% ceramic microparticles [5].

Because of their high mechanical strength, acetal polymers are utilized in a variety of industries like in the manufacturing of structural components (handles, slings) and driving elements (rollers, shafts, gears, etc.). Furthermore, they are frequently discovered in automobiles, office electronics, and home appliances [6]. The biocompatibility of acetal plastic was tested on the Delrin® material from DuPont [7], which was commercialized in 1960. Because of this property, acetal has been applied to medical specialties including orthopedics and cardiac surgery, where it functions well in prosthetic hip or collarbone replacements and heart valves [7,8,9]. Acetal polymers are utilized in dentistry for implant prosthetics, surgery, and orthodontics (as retainers) [10]. Additionally, they are being utilized more and more in dental prostheses to create fixed dentures, serve as the foundation for frame dentures, and serve as clasps made of acetal that are either pink or the natural color of the teeth, depending on whether the clinical crown of the tooth needs to be optically shortened or lengthened [11,12,13,14].

PEEK was developed by a group of English scientists in 1978, later commercialized for industrial applications in 1981 [15], and in 1998, it was recommended as a material for biomedical applications by Invibio Ltd. (Thornton-Cleveleys, UK). In the same year, Victrex PEEK (London, UK) introduced PEEK-OPTIMA for long-term implant applications [16,17]. PEEK, together with polyetherketoneketone (PEKK), belongs to a very large family of polyaryletherketones (PAEK), i.e., high-temperature thermoplastics that, thanks to their wide range of operating temperatures, are characterized by stiffness, high mechanical strength, and resistance to hydrolysis [18]. Thanks to these features, they are used in the aviation industry, automotive industry, semiconductor technology, and medicine [19]. Up to 335 °C, PEEK exhibits exceptional thermal stability and is stiff and radiopaque [20]. At ambient temperature, the solubility is 0.5% by weight in water [16,17,21,22,23]. Its modulus of elasticity (in tension and compression) is 3–4 GPa [24,25], which is comparable to the human cortical bone’s modulus of 7–30 GPa [26,27], but not exactly the same. Its flexural modulus ranges from 140 to 170 MPa. Because of its high strength parameters [28] and other characteristics, PEEK is also used to make a variety of structural components, including pump and valve parts, piston rings, and gears [29]. It is an alternative to metals like titanium because, in comparison to other metal alloys, it is robust, stiff, lightweight, and non-toxic and has a lower modulus of elasticity, which lessens the degree of stress shielding that is frequently seen in titanium-based metal implants [30,31]. In comparison to titanium implants, the results of laboratory testing on PEEK implant prototypes demonstrated a better distribution of occlusal forces surrounding the implant [31,32]. PEEK material has additional benefits over titanium when it comes to producing implants. These benefits include a lower coefficient of friction, superior resistance to abrasive wear, shock absorption capacity, and the absence of metallic color, which greatly enhances the aesthetics of PEEK implants [33]. PEEK implants also have the benefit of being observable under X-rays, which allows assessing the tightness of the prosthetic reconstruction. Since it is not ferromagnetic, patients with PEEK prosthetic restorations can undergo complete diagnostic imaging, including magnetic resonance imaging, without running the risk of heating up these components [16,25,30,32,34,35].

The use of PEEK in medical applications is not limited only to bone implants, but can also includes its use in dental prosthetics as a foundation for both fixed prosthetic restorations (single crowns, conventional bridges, Maryland adhesive bridges) and removable restorations (skeletal dentures, foundations for overdentures based on beam or telescopic crowns) [36]. In conservative dentistry, it can also be used for indirect restorations (inlay; onlay; overlay) [37]. Compared to metals used in dentistry, it is characterized by lower density, greater aesthetics, biocompatibility, and the lack of ferromagnetic properties [38].

The aim of the study was to examine the performance of Biocetal and BioHPP as two competing materials in dental prosthetics. Both materials are available as commercial products with the data sheets describing their physical properties. They are also widely used in prosthetic restoration applications; however, the choice between them is not easy for dentists in individual clinical situations and cases with specific clinical requirements. Biocetal is more suitable for temporary cases, where ease of processing and rapid production are important; however, its application for long-term restorations is currently not recommended, especially in the molar area. BioHPP, on the other hand, is dedicated to long-term solutions, especially in the posterior teeth area, where material resistant to high mechanical loads and abrasion is necessary. BioHPP offers better aesthetic properties, especially in the case of full-ceramic restorations, and also has better translucency, which makes it more similar to a natural tooth. Biocetal does not reach such high aesthetic standards; however, it has very good biocompatibility and is also used in conservative dentistry, so it may be more suitable for patients with greater requirements in terms of the body’s reaction to foreign materials. BioHPP is also a biocompatible material, but due to its higher mechanical properties and material specificity, it may require more caution when selected for patients with certain health problems. There are many aspects to be considered by the doctors, and many cautions, also from the economic standpoint, as BioHPP-manufactured prosthetic products are significantly more expensive. For that reason, a wide-ranging and comprehensive characterization of the physicochemical and mechanical/functional properties of these two widely used prosthetic materials would provide valuable and practical knowledge to dentists, prosthodontists, and medical professionals and facilitate final decisions. It is true that both materials have also been the subject of several scientific publications, although most of these have been primarily reviews [39,40,41,42].

In the presented study, the materials were processed by injection molding into test samples and subjected to mechanical and tribological characterization, typically for material research, in order to compare their properties directly. The obtained results are intended to reveal the strengths and weaknesses of the applications of both of, which may be important in dentists’ decisions about which material will better fulfill its functions in individual clinical cases. To the best of our knowledge, such comprehensive characterizations and comparisons of Biocetal and BioHPP have not been reported yet.

## 2. Materials and Methods

In our studies, the following materials were subjected to characterization: Biocetal produced by ROKO Dental Systems, Częstochowa, Poland, and BioHPP produced by BredentGmbH, Senden, Germany. The tested samples had the dog-bone shapes in accordance with the PN-ISO 37:2005, type 3 standard [43]. The scheme of the specimen design is presented in the Supplementary Information (SI, Figure S1). The modification of the samples in our own study consisted in extending the gripping part in order to mount them in the jaws of the testing machine.

All samples were prepared in the injection molding process using a laboratory scale injection machine Boy 15 (Dr Boy, Neustadt-Fernthal, Germany) and a 10-cavity injection mold. The processing parameters are collected in Table S1. Both materials, in the form of granules, were dried prior to processing at 80 °C for 12 h to eliminate moisture and prevent polymer degradation. When solidified in the mold, the samples were ejected and separated into pieces. Before every physicochemical and mechanical property test, injected samples of both materials were identically treated. As a result, it was decided that sample processing had no bearing on the variations in outcomes between materials.

### 2.1. FTIR Analysis

Fourier-transform infrared spectroscopy in ATR mode was performed using a Tensor-27 spectrophotometer (Brucker, Ettlingen, Germany). The spectra were recorded within the wave number range of 4000 to 400 cm^−1^.

### 2.2. Hardness Test

The hardness of Biocetal and BioHPP samples was measured using the Shore durometer, scale D (Matbor, Sosnowiec, Poland), with 5 kg weight according to the PN-ISO 868 standard [44]. Two pieces of samples were stacked together to obtain 4 mm in thickness. Ten tests were performed for every material, and the results were read from the scale after 15 s.

### 2.3. Impact Strength Test

The Charpy method was used to test the impact strength of samples made of thermoplastic. The study used a special Charpy-type hammer composed of a base and two pillars in which a pendulum with a hammer was mounted. A hammer with an energy of 5 J was used for the test. Ten unnotched samples made of Biocetal material and 10 samples made of BioHPP were tested. The hammer blade speed was 2.9 m/s. The falling hammer breaks the specimen, which absorbs the appropriate amount of energy, and the pointer shows the work used to break the sample.

The impact strength of samples without notches (*a_n_*) is described by the following formula:

$$a_{n}=\frac{A_{n}}{b\cdot h}\left[\mathrm{kJ}/m^{2}\right]$$

where:

*A_n_*—work consumed for dynamic fracture of a sample without a notch, related to 1 m^2^ of cross-section at the point of fracture,

*b*—sample width [m],

*h*—sample height [m].

According to the standard, bars with a width of 10 mm are used. However, due to the use of our own universal test shapes, the sample width was 4 mm, and the thickness was 2 mm. The markings were made on the measuring part of the fittings, which is acceptable because the fracture work refers to the actual cross-section of the sample.

### 2.4. Tensile Strength Test

Tensile strength testing was performed using a universal testing machine (ElectroPuls E10000, Instron, Darmstadt, Germany) equipped with an Instron AVE2 video extensometer (Instron, Darmstadt, Germany). The samples 2 × 4 mm^2^ in cross-section were first deformed up to 1% at a speed of 1 mm/min in order to calculate the elastic modulus, and then, at 5 mm/min up to the sample break. Eight samples from Biocetal and eight from BioHPP were used for the study.

Tensile strength (*σ_m_*) is the stress corresponding to the highest tensile force *F_m_* related to the original cross-sectional area of the sample. This is described by the formula below:

$$\sigma_{m}=\frac{F_{m}}{S_{0}}\left[\mathrm{MPa}\right]$$

where:

*F_m_*—value of the greatest (maximum) force [N],

*S*_0_—value of the initial cross-sectional area of the sample [mm^2^].

An important parameter is the stress that acts on the sample at the yield point (*σ_y_*), but the stress at break (*σ_B_*) is also determined.

The yield point (*σ_y_*) is the stress at which the material becomes permanently deformed, i.e., irreversible, when microscopic plastic deformations begin to occur. The conventional criterion for determining this limit is a permanent relative deformation of 0.002. When describing the yield strength parameter, it is important at what stress and relative elongation it occurs. The elastic limit is the stress beyond which the material does not return to its original shape after the stress is removed. The yield strength is described by the following formula:

$$\sigma_{y}=\frac{F_{y}}{S_{0}}\left[\mathrm{MPa}\right]$$

where:

*F_y_*—the value of the force corresponding to the conventional yield point [N],

*S*_0_—the value of the initial cross-sectional area of the sample [mm^2^].

Relative elongation (Ɛ) is the ratio of the increase in length to the initial length of the measurement section, expressed in percent (%). It is expressed by the formula below:

$${Ɛ}_{x}=\frac{{\Delta l}_{x}}{l_{0}}*100\%$$

where:

Ɛ_x_—relative elongation,

Δ*l_x_*—absolute deformation [m],

*l*_0_—initial length of the measurement section [m].

The relative elongation can be measured at the yield point (Ɛ_y_), at break (Ɛ_B_), and at the maximum tensile stress (Ɛ_M_), which corresponds to the elongation at the point corresponding to the tensile strength.

The elastic modulus (E) is also called the modulus of linear deformation or the coefficient of longitudinal elasticity. This value determines the elasticity of the material when stretched and compressed. It expresses the dependence of the relative linear deformation (Ɛ) of the material on the stress (σ) that occurs in it. The deformations of the samples during static tensile strength testing were analyzed using an Instron AVE2 video extensometer (Instron, Darmstadt, Germany).

### 2.5. Flexural Strength Test

The Autograph AG-Xplus universal testing device (Shimadzu, Kyoto, Japan) was used to conduct the test. To test the strength, 5 samples each from Biocetal and BioHPP were utilized. They were stretched with a 32 mm support spacing at a rate of 1 mm/min. Once the test reached its peak stress, it was terminated. The samples were not destroyed, but were permanently deformed.

When bending materials, the maximum bending stress and the relative strain at the maximum stress are determined.

### 2.6. Abrasive Wear Testing

The aim of the tribological test was to analyze the susceptibility to abrasive wear of Biocetal and BioHPP under dry friction conditions and in the presence of a liquid imitating human saliva (“wet”), taking into account factors affecting the material’s susceptibility to wear. The speed of surface wear was compared. The tests were performed on a tribometer from CSM Instruments SA. In the test, a spherical, ceramic counter sample was pressed against the sample with a precisely known force of 2N. The counter sample was mounted on a rigid holder, which is a frictionless force transmitter. The coefficient of friction is determined by measuring the deformation of the flexible arm. The abrasive wear rate of the sample is calculated from the volume of the material that was abraded during the test (wear volume).

As a result of the study, the wear rate was determined. One way to determine it is to calculate the appropriate consumption rate *K*, based on the formula below:

$$K=V/(F_{n}*s)\left[m^{3}/N*m\right]$$

where

*K*—consumption rate [m^3^/N∗m],

*V*—wear trace volume [m^3^],

*F_n_*—normal load [N],

*s*—total friction path [m].

The value of the normal load was selected based on the mechanical properties of the two friction materials, assuming point contact (ball as counter sample), based on the Hertz equation (Table 1):

Process parameters were as follows: rotational speed of the measurement sample, 60 rpm; normal load, 2 N; total sliding distance, 5000 m; counter sample radius, 2.997 mm.

The volume of the wear trace was calculated from the following formula:

$$V=A_{cs}*\pi *D$$

where:

*A_cs_*—cross-sectional area [µm^2^],

π—PI number (~3.1415),

*D*—diameter of the worn circle [mm].

The outer and inner diameters of all samples were measured using a digital camera (Keyence, Osaka, Japan). The cross-sections of wear traces were measured using a digital microscope from the same company. Measurements were also made on an optical coordinate measuring machine STRATO-Apex 574 CNC CMM (Mitutoyo, Kawasaki, Japan) with the possibility of measuring topography using the interference method.

The study was performed on three samples of BioHPP and Biocetal in “dry” conditions and two samples of each material in “wet” conditions using the artificial saliva available as Kserostemin (Aflofarm, Pabianice, Poland).

### 2.7. Statistical Analysis

The results obtained are presented in tables and graphs for each experiment and material. For each variable, the number of samples used, the minimum and maximum values of the measurements, the median, the first and third quartiles, the mean, the standard deviation, and the standard error are presented. To assess the normality of the distribution of the studied variables, the Shapiro–Wilk test was used. If deviations from the normal distribution were found, the variable was assessed for outliers using a Q–Q chart analysis and the Grubbs test. If there was no significant deviation in the distribution of variables from the normal distribution in both groups, the Student’s t-test was used for comparisons between groups. The assumption of homogeneity of variances was verified using the Levene test, and if it was not met, the Student’s t-test with Welch’s correction was used. The analysis was performed in the R language in the Rstudio environment using the tidyverse package. The results of statistical analyses were considered statistically significant at *p* < 0.05. The paper contains the main results of the individual studies, with detailed statistical analysis provided in the SI (Tables S2–S4).

## 3. Results

### 3.1. FTIR Analysis Results

The ATR-FTIR spectrum recorded for Biocetal and presented in (SI, Figure S2) shows strong absorption bands at 891 and 1088 cm^−1^, which are attributed to the C-O-C stretching bonds, as well as the bands at 628, 1238, and 2912 cm^−1^ corresponding to CH_2_ stretching, and at 1470 cm^−1^ to CH_2_ bending bonds. According to the literature [45,46], all these absorption bands are characteristic of polyoxymethylene. In turn, the spectrum recorded for BioHPP (SI, Figure S2) reveals a variety of absorption bands within the wave number range 1800–600 cm^−1^ that are characteristic of poly(ether ether ketone) [47,48]. The band at 1652 cm^−1^ is attributed to the stretching vibration of C=O bond, and the phenyl ring stretching bands appear at 1593, 1486, and 1410 cm^−1^. Next, the bending motion band of C-C and (=O)-C group is detectable at 1305 cm^−1^, and at 1277, 1216, and 1184 cm^−1^, the bands attributed to asymmetric stretching of the diphenyl ether group can be distinguished. Moreover, in the 1200–600 cm^−1^ region, the bands attributed to phenyl ring C-H bond deformations can be found. Thus, the obtained spectra confirmed that POM and PEEK were the main polymer materials in Biocetal and BioHPP, respectively.

### 3.2. Shore’s Hardness Test Evaluation

A moderately higher Shore’s hardness of the BioHPP material was observed compared to Biocetal. The average hardness of Biocetal was 78 ± 1 ShD, while the average hardness of BioHPP was 82 ± 2 ShD.

### 3.3. Impact Strength Test Evaluation

**Figure 1:**
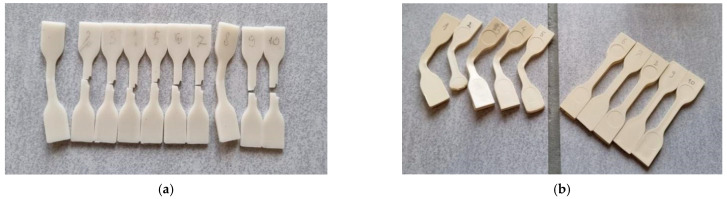
Samples of both materials after impact testing: Biocetal (**a**) and BioHPP (**b**).

In the Charpy impact strength test, BioHPP did not break in any of the 10 tests, despite the use of a hammer with the highest energy (5 J), as shown in Figure 1. The average impact strength of Biocetal—i.e., the impact energy absorbed during the fracture of the fitting in relation to the initial cross-sectional area of the fitting, was on average 147 ± 14 kJ/m^2^ (Table 2).

### 3.4. Tensile Strength Test Evaluation

**Figure 2:**
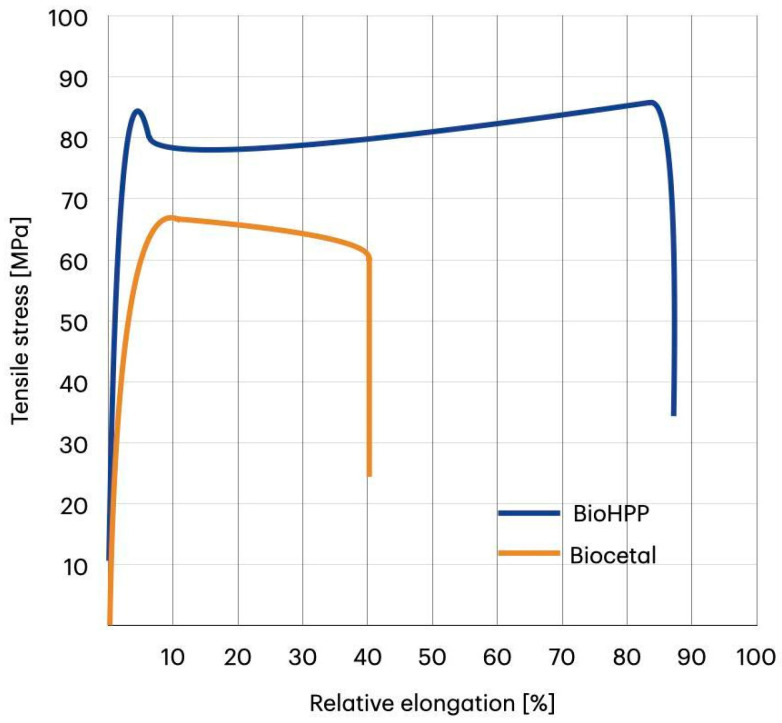
Representative stress–strain characteristics during static uniaxial stretching.

The mechanical parameters of both materials obtained in static uniaxial tensile tests are presented in Table 3, whereas the stress–strain curves are visible in Figure 2. The stress–strain characteristics show that both materials have a completely different way of deforming. BioHPP deforms longer before breaking and “flows” longer—i.e., its elongation increases without an increase in stress. This may be the result of the presence of filler particles. The toughness, defined as the ability of a material to absorb an energy and deform plastically without fracturing, reflected by the area under the stress–strain curve, is significantly higher for BioHPP.

However, the elongation of BioHPP at the yield point is significantly lower than the elongation of Biocetal. The averages were 4.04 ± 0.1% for BioHPP and 11.7 ± 0.3% for Biocetal. The BioHPP stress value at the yield point was significantly higher than the Biocetal stress value. The averages were 84.8 ± 1.9 MPa for BioHPP and 67.0 ± 0.4 MPa for Biocetal. The average stress at fracture of BioHPP was higher than that of Biocetal and amounted to 69.1 ± 16 MPa, while that of Biocetal was 57.7 ± 3 MPa. From a material point of view, this difference is significant, but from a statistical analysis, the difference in these values is not statistically significant. The relative elongation of BioHPP at fracture was significantly higher than that of Biocetal. The averages were 87.3 ± 7.09% for BioHPP and 45.4 ± 14.1% for Biocetal. The elastic modulus of BioHPP was significantly higher compared to Biocetal. It was on average 5.34 ± 0.32 GPa for BioHPP and 3.11 ± 0.14 GPa for Biocetal.

### 3.5. Flexural Strength Test Evaluation

**Figure 3:**
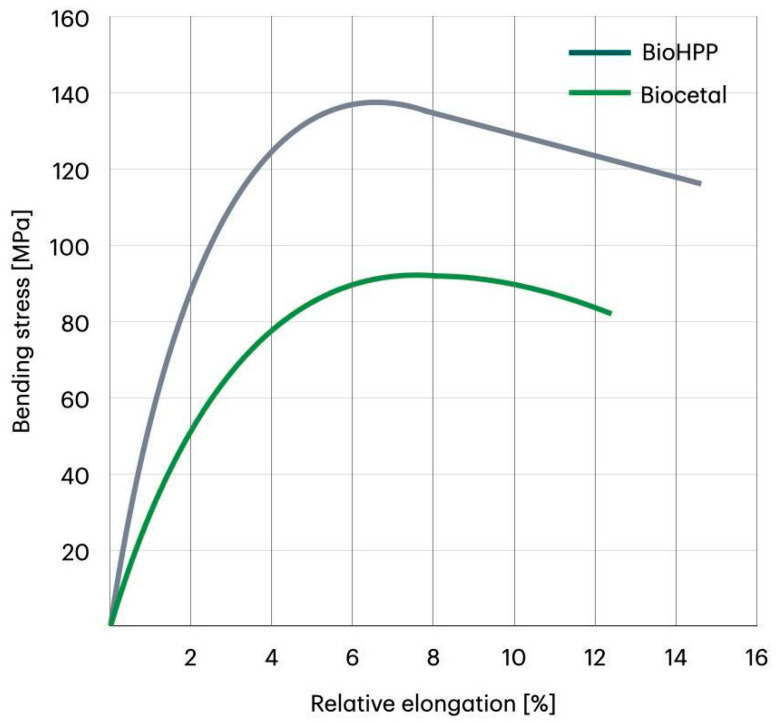
Representative stress–strain characteristics during a static bending test.

The stress–strain characteristics obtained during bending tests are presented in Figure 3. Data are collected and compared in Table 4.

As one can see, BioHPP is characterized by a significantly higher maximum stress in the bending test if compared to Biocetal. The average stress value was 137 ± 2.09 MPa for BioHPP and 93 ± 0.3 MPa for Biocetal. Significantly higher relative strain at maximum stress was observed for Biocetal than for BioHPP. The average relative strain at maximum stress for Biocetal was 7.73 ± 0.14%, while for BioHPP, it was on average 6.29 ± 0.22%.

### 3.6. Abrasive Wear Test Evaluation

**Figure 4:**
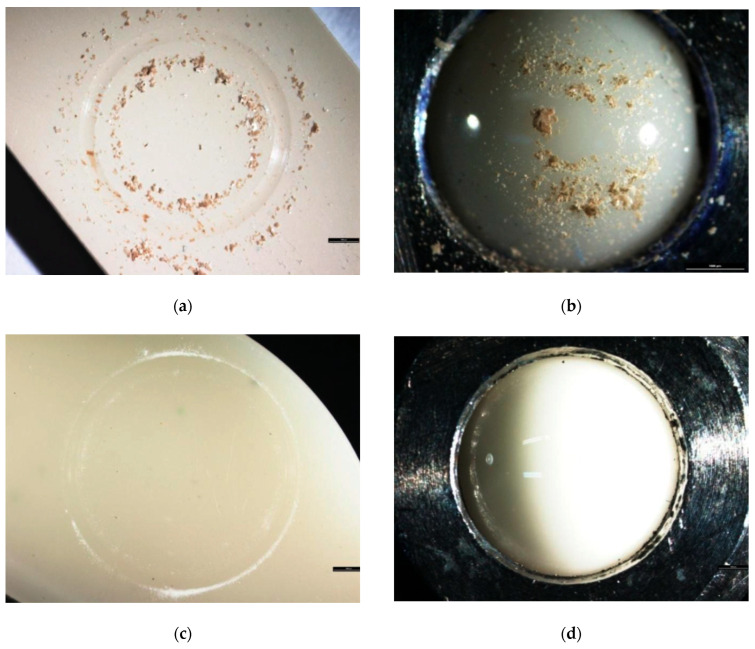
A microscopic image of the BioHPP sample (**a**) and the ceramic counter sample (**b**), as well as an image of the Biocetal sample (**c**) and the ceramic counter sample (**d**) taken immediately after the test.

The abrasive wear tests showed a large wear mark at the point of contact with the counter sample for the BioHPP material. In the microscopic analysis of the sample, material particles “pulled out” by friction were visible, accumulating in larger clusters, both on the sample and on the ceramic counter sample (Figure 4a,b).

In the case of Biocetal, a trace of wear was also visible, but the particles were much finer and their amount was smaller. Moreover the powder formed as a result of friction was more uniform compared to BioHPP (Figure 4c,d).

**Figure 5:**
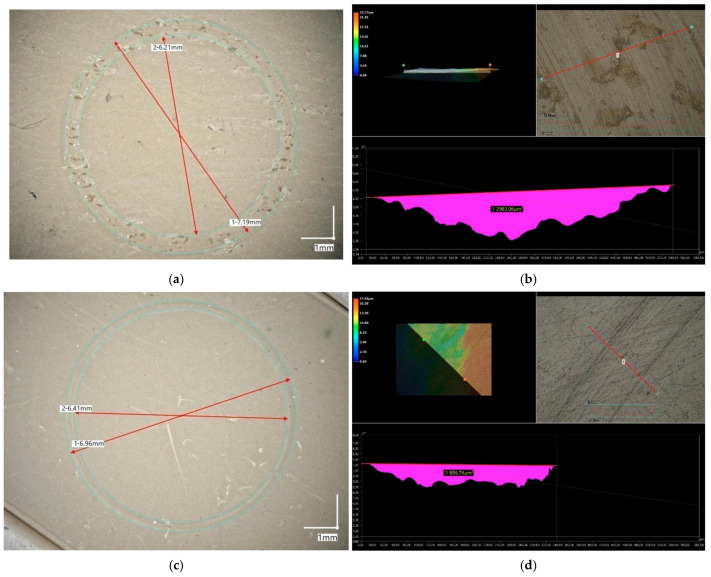
A microscopic image of a wear trace (**a**) and an image of the surface area [µm^2^] (**b**) of a representative BioHPP sample and an image of a wear trace (**c**) and an image of the surface area [µm^2^] (**d**) of a representative Biocetal sample, the diameters of which were used to calculate the wear rate (K) in the “dry” test.

A microscopic analysis of the abrasion sites was performed to measure the cross-sectional area of the defect after testing in “dry” conditions (Figure 5, SI Figure S3). The measurements were made on several samples and in several places. The averaged result was used to calculate the abrasive wear coefficient. Cross-sections of wear traces were measured using a digital microscope.

**Figure 6:**
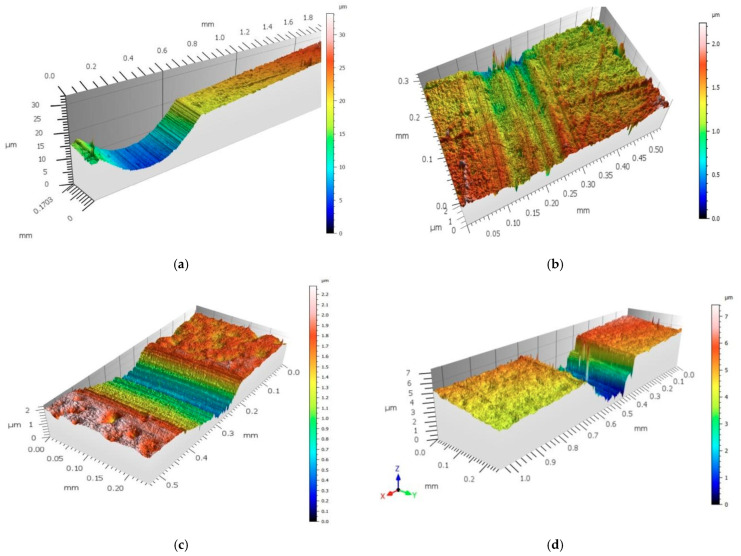
Analysis of the surface profile of the BioHPP sample during dry (**a**) and “wet” (**b**) testing and the surface of the Biocetal sample during “dry” (**c**) and “wet” (**d**) testing at the point of clash.

Topography measurements using the interference method performed on the STRATO-Apex 574 CNC CMM optical coordinate measuring machine (Mitutoyo, Kawasaki, Japan) are shown in Figure 6.

The abrasive wear results are included in Table 5. In “wet” abrasive wear, BioHPP shows less abrasion than Biocetal, 6.2652 × 10^−17^ m^3^/Nm and 1.5075 × 10^−15^ m^3^/Nm, respectively. In turn, in “dry” abrasive wear, Biocetal shows less abrasion than BioHPP, 2.3514 × 10^−15^ m^3^/Nm and 6.9887 × 10^−15^ m^3^/Nm, respectively.

## 4. Discussion

One of the most crucial aspects of analyzing the utility of materials is figuring out how different dental prosthetic materials differ from one another in terms of their physicochemical and mechanical properties. A dentist must be able to choose a material that is both resistant to the conditions in the oral cavity and aesthetically pleasing when making prosthetic restorations in a particular clinical case [49]. The price of the material used also has a major impact on the final price of the prosthetic treatment, which in turn is of interest for the patient. In our studies, two commercially available biomaterials, Biocetal and BioHPP, were subjected to broad comparative characterization as regards their mechanical and tribological performance in order to highlight their strengths and weaknesses. Although both materials were purchased as commercial products, their chemical structures were confirmed by FTIR analysis.

In the hardness test, the BioHPP material’s hardness (82 ± 2 ShD) was found to be only slightly higher than that of Biocetal (78 ± 1 ShD). PLASTOSEAL^®^ presented the parameters of the acetal (polyoxymethylene) they use. The hardness of their material according to the Shore scale was 82 ± 3 ShD. Although the results of our own investigation can be compared to this one, it is important to remember that commercial products may vary. The average energy absorbed by the Biocetal samples in the impact strength test was 146 ± 14 kJ/m^2^, which was sufficient to break them. The BioHPP samples remained intact after being struck with a hammer with an energy of 5 J, suggesting that they had a higher hardness than the Biocetal samples. As demonstrated by the Ensinger company’s product, TecaPEEK natural (around 120 kJ/m^2^), the PEEK homopolymer (poly(ether ether ketone)) has an extremely high impact resistance. The impact strength resulting from the formation of areas of decreased cohesiveness should be lessened by further dispersing different particles within the material to lessen its homogeneity. On the other hand, greater impact resistance was obtained by using ceramics in the BioHPP composite. From the perspective of patients using prostheses, higher material hardness is crucial, as are other mechanical characteristics, including impact strength and resistance to deformation. Clinically, these characteristics result in a notable resistance of BioHPP dentures against breakage and damage, particularly when biting into tough foods or inadvertently falling from a height. In order to create composites and increase the performance parameters of the material, various modifications are used, such as the use of a polyether ether ketone matrix, which allows the dispersion of glass [50] and carbon fibers (CFR-PEEK) [51,52]. The addition of carbon fibers to PEEK increases its strength and dimensional stability, hardness, resistance, and bending strength [51].

In the remaining mechanical tests, such as tensile strength and three-point bending, various parameters were considered, such as stress and elongation at yield, stress and elongation at fracture, and elasticity modulus. All these parameters highlighted very high stiffness and resistance of the BioHPP material to external forces and the method of deformation. However, from a practical point of view, the most important parameters are those recorded at the yield point.

The maximum value of elongation a material sample can attain during deformation when permanent damage to the material occurs is known as relative elongation at break. Compared to BioHPP, which had a relative elongation of 87.3 ± 7.09% at fracture, Biocetal had a substantially lower relative elongation of 45.4 ± 14.1%, and there was a notable variation in the outcomes (from a minimum of 29.26% to a maximum of 66.97%). Since acetal plastic is a homopolymer, fillers—which may have an impact on local crack resistance—are not likely to be encountered. However, elongation at yield seems to be more important, as it is the value to which a material can be stretched without permanent deformation. The greater the elongation at the yield point, the more flexible the material is. It is obvious that yield stress is significantly higher for BioHPP (84.8 ± 1.9 MPa vs. 67.0 ± 0.4 MPa); however, elongation of Biocetal at yield was 11.7 ± 0.3%, and the elongation of BioHPP was 4.04%. This means that Biocetal is a more flexible material than BioHPP within the initial elastic deformation region, which makes it better suited for applications where greater flexibility is required. This is one of the features that speaks for the superiority of Biocetal over BioHPP in the production of dentures, as a restoration made in this way will be more resistant to accidental cracks during its use.

On the other hand, BioHPP is more suitable for applications needing high mechanical strength because it can transfer stress more effectively than Biocetal and is more resistant to plastic deformation. The stress value at which a crack appears and the material fails is known as the fracture stress. The more resistant a material is to mechanical harm, the higher this value is. In our investigation, BioHPP had a stress value at fracture of 69.1 ± 16 MPa, whereas Biocetal had a stress value of 57.7 ± 3 MPa. There is a discernible variation in materials, although it is not statistically significant.

Elastic modulus is a measure of a material’s stiffness, i.e., its ability to maintain its shape under load. The higher the modulus, the stiffer the material and the less susceptible to deformation it is. The value of Biocetal's elastic modulus in our own study was on average 3.11 ± 0.14 GPa, which is significantly lower than the elastic modulus of BioHPP, which was on average 5.34 ± 0.32 GPa. Another parameter proves that BioHPP is a more resistant material than Biocetal and is better suited for applications where greater stiffness is required, such as crowns or prosthetic bridges. Sandler et al. [53] measured the static uniaxial stretching of PEEK samples. The head speed was initially set to 0.5 mm/min in the strain range of 0–0.25%, and then the speed was increased to 10 mm/min until the sample broke. The resulting stress at the yield point of the pure PEEK matrix was approximately 80 MPa, and the elastic modulus was 4 ± 0.1 GPa, which, despite the lack of a microfiller, is a result similar to the values obtained in our own study.

The mechanical characteristic that establishes a material’s resistance to fracture under load in the three-point bending test is the maximum stress. When the material is deformed in this test, it reaches its maximum stress value. A material’s resistance to fracture and suitability for applications requiring high mechanical strength increase with its maximum stress. Our own research revealed that BioHPP was a more resilient material, with an average maximum stress value of 137 ± 2.09 MPa, compared to 93 ± 0.3 MPa for Biocetal. PEEK samples from two manufacturers, VESTAKEEP® (M4 R) (Evonik Industries, Germany) and PEEK-OPTIMA^®^ (LT1) (Invibio Ltd., UK), were subjected to a three-point bending test by Schwitalla et al. [54]. Maximum stresses of 170.37 ± 19.31 MPa and 182.91 ± 12.59 MPa, respectively, were reached by the materials. There is a variation in the results because the BioHPP material utilized in our investigation contained a ceramic filler, whereas that in the study by Schwitalla et al. did not. While the values in the Schwitalla et al. study varied greatly (standard deviation equal to 19.31 MPa), the maximum stress levels in our investigation did not deviate significantly from the average value (standard deviation equal to 2.09 MPa).

The amount of strain a material experiences in a bending test when it achieves its highest stress is known as relative strain at maximum stress. The smaller the relative deformation, the higher the modulus of elasticity the material has and the less deformable it is, which may affect its strength and stability. BioHPP exhibited a notably reduced relative strain (6.29 ± 0.22%) at maximum stress compared to Biocetal (7.73 ± 0.14%). This means that BioHPP is a material with a higher modulus of elasticity and has a lower ability to deform than Biocetal. Actually, this is in agreement with tensile test results, once again confirming the more flexible behavior of Biocetal.

To summarize the tests of the strength of materials under uniaxial tensile and three-point bending, undoubtedly BioHPP is a high-performance composite material with higher strength and is less susceptible to destruction. Nevertheless, Biocetal also reveals relatively high mechanical parameters and can be considered for applications where greater flexibility is required. It is difficult, however, to start a discussion with other authors due to the lack of similar studies in the available literature regarding exactly the same materials, which makes it impossible to verify the obtained results with data from the literature.

Considering tribological performance, one can say that both materials suffered wear abrasion from friction with a ceramic counter sample under “dry” and “wet” conditions. In both situations, the volume of the abrasion trace was used to determine the material wear volume (V) and wear rate (K). In the “dry” abrasive wear test, Biocetal demonstrated significantly better resistance. The surface of Biocetal exhibited fine dust, a sign of the material’s homogeneity. In the case of BioHPP, worn-out material clusters were evident following the abrasive wear test. This suggests the existence of detachable agglomerates of ceramic particles that were rubbed into the material further, increasing the friction of the ceramics against the sample. This is a disadvantage when using this material for dentures worn by people who produce insufficient saliva, as it can cause faster wear of the dentures. Moreover, both wear parameters, volume and rate, were significantly higher for BioHPP under “dry” conditions (V: 6.99 × 10^−11^ vs. 2.35 × 10^−11^, K: 6.99 × 10^−15^ vs. 2.35 × 10^−15^, respectively), which clearly proves that PEEK composite is less resistant and undergoes unfavorable abrasive wear when in friction contact with harder materials.

Water evaporation throughout the test in artificial saliva altered the content and viscosity of the water-based product Kserostemin (Aflofarm, Pabianice, Poland). Furthermore, a change in conditions during the “wet” test was also impacted by the preparation’s elevated temperature during the friction test. The Kserostemin preparation’s water content might potentially seep into the samples. This affected the friction coefficient results as well as the instability of the test conditions. Nonetheless, it is reasonable to regard the acquired wear volume and rate data as trustworthy.

Compared to dry testing, wet testing in artificial saliva revealed a decreased rate of abrasive wear of both materials: for BioHPP, the wear rate in the “wet” test was 6.2652 × 10^−17^ m^3^/Nm, which was up to 111.55 times lower than that in the “dry” test. In the case of Biocetal, the “wet” test wear rate was 1.5075 × 10^−15^ m^3^/Nm, showing that the wear rate in the artificial saliva preparation was only 1.56 times lower than in “dry” conditions.

Because of the previously described fractured clusters of ceramic filler, which continued to grind against the BioHPP sample in “dry” settings, causing even higher abrasion, there was a difference in the wear rate between BioHPP and Biocetal in both wet and dry conditions. Any abraded filler clusters were washed away by fresh salivary segments in the artificial saliva preparation (i.e., under conditions akin to those in the oral cavity).

Comparing identical conditions for both materials, in the “dry” test, Biocetal showed 2.97 times less abrasive wear compared to BioHPP, while in the conditions of artificial saliva—the “wet” test, Biocetal showed 24.06 times less abrasive wear than BioHPP.

## 5. Conclusions

The presented results for mechanical and tribological performance allow a direct comparison of two polymer materials, dedicated to use in dental prostheses, in order to reveal the strengths and weaknesses of their applications. That may be important in dentists’ decisions regarding which material will better fulfill its functions in individual clinical cases. Our studies confirmed what is stated by their manufacturers, namely, that both BioHPP and Biocetal are classified as high-performance materials. In particular, BioHPP, a PEEK-based composite containing ceramic microfiller, is characterized by very high mechanical parameters but also a relatively high market price. However, the performed experiments showed that in some characteristics, BioHPP reveals drawbacks: specifically, (1) it is subject to significant abrasive wear in “dry” conditions and (2) reveals a smaller range of elastic deformation at yield. Both of these characteristics may lead to the poorer use of prostheses, first, due to faster wear of the material in elderly people producing less saliva (the main group using prostheses), and second, due to poorer mechanical strength and resistance to cracks during long-term use of the prosthesis.

In vitro mechanical tests on Biocetal and BioHPP have limitations, as they do not fully reflect the complex environment of the oral cavity and all the processes occurring therein. The samples used in the study, despite being made in the laboratory using the same method, do not reflect the anatomical diversity of patients’ teeth, whose surfaces are not smooth like the surfaces of the samples made and are subject to forces distributed in different directions. The results from our study are also short-term results that did not reflect the long-term durability of materials, such as wear, because we did not have the appropriate equipment for that type of study. In future studies, the authors also plan to investigate the biocompatibility of both materials as well as their physicochemical parameters, which are difficult to include in one article due to the large number of tests involved.

## Figures and Tables

**Table 1:** Determination of abrasive wear test parameters.

**Sample: Biocetal**	**Counter Sample: Al_2_O_3_**
E-modulus ~3 GPa; Poisson’s ratio 0.35; Radius of curvature: 0 for a flat sample	E-modulus ~370 GPa; Poisson’s ratio 0.22; Radius of curvature: 3 mm (0.003 m) for a ball diameter of 6 mm
For 1N load—average contact pressure: 0.04 GPa (40 MPa), max contact pressure 0.06 GPa (60 MPa) For a 2N load—average contact pressure: 0.05 GPa (50 MPa), max contact pressure 0.08 GPa (80 MPa) Maximum tensile stress 67—70 MPa	
**Sample: BioHPP**	**Counter Sample: Al_2_O_3_**
E-modulus ~5.5 GPa; Poisson’s ratio 0.37; Radius of curvature: 0 for a flat sample	E-modulus ~370 GPa; Poisson’s ratio 0.22; Radius of curvature: 3 mm (0.003 m) for a ball diameter of 6 mm
For a 1 N load—average contact pressure: 0.06 GPa (60 MPa), max contact pressure 0.09 GPa (90 MPa) For a 2 N load—average contact pressure: 0.08 GPa (80 MPa), max contact pressure 0.12 GPa (120 MPa) Stresses at the yield point 85–87 MPa; Maximum stresses not greater than 90 MPa	

**Table 2:** Summary of the obtained mechanical test results.

Material	Medium Hardness [ShD]	Medium Impact Strength [kJ/m^2^]
Biocetal	78 ± 1	147 ± 14
BioHPP	82 ± 2	No breakage

**Table 3:** Summary of the mechanical parameters obtained in the tensile tests of materials.

Material	Relative Elongation at Yield Point [%]	Stress at Yield Point [MPa]	Stress at Break [MPa]	Relative Elongation at Break [%]	Elastic Modulus (0.05–0.25) [GPa]
Biocetal	11.7 ± 0.3	67.0 ± 0.4	57.7 ± 2.9	45.4 ± 14.1	3.11 ± 0.14
BioHPP	4.04 ± 0.13	84.8 ± 1.9	69.1 ± 16	87.3 ± 7.1	5.34 ± 0.32

**Table 4:** Summary of the test results obtained in the material bending test.

Material	Maximum Tension [MPa]	Relative Strain at Maximum Stress [%]
Biocetal	93 ± 0.3	7.73 ± 0.14
BioHPP	137.00 ± 2.09	6.29 ± 0.217

**Table 5:** Abrasiveness results for Biocetal and BioHPP samples, depending on the test conditions.

Material and Test Conditions	Biocetal “Dry”	Biocetal “Wet”	BioHPP “Dry”	BioHPP “Wet”
V—wear volume [m^3^]	2.3514 × 10^−11^	1.5075 × 10^−11^	6.9887 × 10^−11^	6.2652 × 10^−13^
K—wear rate [m^3^/Nm]	2.3514 × 10^−15^	1.5075 × 10^−15^	6.9887 × 10^−15^	6.2652 × 10^−17^

## Data Availability

All the raw data are available from the corresponding author on reasonable request due to privacy.

## Supplementary Materials

The following supporting information can be downloaded at: https://www.mdpi.com/article/10.3390/ma18030561/s1. Figure S1. Sample design; Figure S2. ATR-FTIR spectra of Biocetal (a) and BioHPP (b); Figure S3. A microscopic image of the wear trace of a representative sample of BioHPP (a) and Biocetal (b), the diameter of which was used to calculate the wear rate (K) in the “wet” test; Table S1. Injection molding parameters; Table S2. Statistical analysis of hardness determination results; Table S3. Statistical analysis of the results of tensile tests; Table S4. Statistical analysis of the results of determining the maximum stress and relative strain in the bending test.

## References

[1] Galindo-Moreno P., Lopez-Chaichio L., Padial-Molina M., Avila-Ortiz G., O’valle F., Ravida A., Catena A. (2022). The impact of tooth loss on cognitive function. Clin. Oral Investig..

[2] Saeed F., Muhammad N., Khan A.S., Sharif F., Rahim A., Ahmad P., Irfan M. (2020). Prosthodontics dental materials: From conventional to unconventional. Mater. Sci. Eng. C Mater. Biol. Appl..

[3] Fueki K., Ohkubo C., Yatabe M., Arakawa I., Arita M., Ino S., Kanamori T., Kawai Y., Kawara M., Komiyama O. (2014). Clinical application of removable partial dentures using thermoplastic resin-part I: Definition and indication of non-metal clasp dentures. J. Prosthodont. Res..

[4] Sullivan K.P., Yin Q., Collins-Wildman D.L., Tao M., Geletii Y.V., Musaev D.G., Lian T., Hill C.L. (2018). Multi-Tasking POM Systems. Front. Chem..

[5] Ramadan R., Elsherbeeny Y., Thabet Y., Kandil B., Ghali R. (2021). Retention of a telescopic overdenture on customized abutments after the simulation of 1 year in function. Dent. Med. Probl..

[6] Gray D., Barraclough O., Ali Z., Nattress B. (2021). Modern partial dentures—Part 2: A review of novel metal-free materials and innovations in polymers. Br. Dent. J..

[7] Fister J.S., Memoli W.A., Galante J.O., Rosteker W., Urban M.R. (1985). Biocompatibility of Derlin 150: A creep—Resistant polimer for total joint prostheses. J. Biomed. Mater. Res..

[8] Maeda M. (1984). Experimental studies on polyacetal composites for joint prosthesis. Nippon. Seikeigeka Gakkai Zasshi.

[9] Kayser M., Seiler H. (1985). Case report on the partial replacement of the clavicle using a polyacetal resin prosthesis. Unfallchirurg.

[10] Kirsch A., Ackermann K.L. (1989). The IMZ osteointegrated implant system. Dent. Clin. N. Am..

[11] Lagemann U., Heinzelmann I. (1997). Azetal—Ein innovativer Werkstoff. Quintessenz Zahntech..

[12] Rutkowski A. (2007). Acetal—Estetyczna alternatywa rozwiązań protetycznych. Nowocz. Tech. Dent..

[13] Sikorska—Bochińska J., Urbanek R. (2005). Elastyczne i sprężyste tworzywo na protezy ruchome i stałe w aspekcie alergii kontaktowej. Twój Prz. Stom..

[14] Ardelean L., Bortun C.M., Podariu A.C., Das C.K. (2015). Thermoplastic Resins used in Dentistry. Thermoplastic Elastomers—Synthesis and Applications.

[15] Staniland P., Wilde C., Bottino F., Di Pasquale G., Pollicino A., Recca A. (1992). Synthesis, characterization and study of the thermal properties of new polyarylene ethers. Polymer.

[16] Kurtz S.M., Devine J.N. (2007). PEEK biomaterials in trauma, orthopedic, and spinal implants. Biomaterials.

[17] Green S. (2015). A polyaryletherketone biomaterial for use in medical implant applications. Chem. Artic. News.

[18] Maloo L.M., Toshniwal S.H., Reche A., Paul P., Wanjari M.B. (2022). A Sneak Peek Toward Polyaryletherketone (PAEK) Polymer: A Review. Cureus.

[19] Alexakou E., Damanaki M., Zoidis P., Bakiri E., Mouzis N., Smidt G., Kourtis S. (2019). PEEK High Performance Polymers: A Review of Properties and Clinical Applications in Prosthodontics and Restorative Dentistry. Eur. J. Prosthodont. Restor. Dent..

[20] Monich P.R., Berti F.V., Porto L.M., Henriques B., de Oliveira A.P.N., Fredel M.C., Souza J.C. (2017). Physicochemical and biological assessment of PEEK composites embedding natural amorphous silica fibers for biomedical applications. Mater. Sci. Eng. C Mater. Biol. Appl..

[21] Stober E.J., Seferis J.C., Keenan J.D. (1984). Characterization and exposure of polyetheretherketone (PEEK) to fluid environments. Polymer.

[22] Searle O.B., Pfeiffer R.H. (1985). Victrex poly(ethersulfone) (PES) and Victrex poly(etheretherketone) (PEEK). Polym. Eng. Sci..

[23] Boinard E., Pethrick R.A., McFarlane C.J. (2000). The influence of thermal history on the dynamic mechanical and dielectric studies of polyetheretherketone exposed to water and brine. Polymer.

[24] Xin H., Shepherd D., Dearn K. (2013). Strength of poly-etherether-ketone: Effects of sterilisation and thermal ageing. Polym. Test..

[25] Schwitalla A., Muller W.D. (2013). PEEK dental implants: A review of the literature. J. Oral Implantol..

[26] Kizuki T., Matsushita T., Kokubo T. (2015). Apatite-forming PEEK with TiO_2_ surface layer coating. J. Mater. Sci. Mater. Med..

[27] Garcia-Gonzalez D., Rusinek A., Jankowiak T., Arias A. (2015). Mechanical impact behavior of polyether-ether-ketone (PEEK). Compos. Struct..

[28] Cheol-Min H., Eun-Jung L., Hyoun-Ee K., Young-Hag K., Keung N.K., Yoon H., Sung-Uk K. (2010). The electron beam deposition of titanium on polyethereethereketone (PEEK) and resulting enhanced biological properties. Biomaterials.

[29] Dua R., Rashad Z., Spears J., Dunn G., Maxwell M. (2021). Applications of 3D-Printed PEEK via Fused Filament Fabrication: A Systematic Review. Polymers.

[30] Toth J.M., Wang M., Estes B.T., Scifert J.L., Seim I.I.I.H.B., Turner A.S. (2006). Polyetheretherketone as a biomaterial for spinal applications. Biomaterials.

[31] Lee W.T., Koak J.Y., Lim Y.J., Kim S.K., Kwon H.B., Kim M.J. (2012). Stress shielding and fatigue limits of poly-ether-ether-ketone dental implants. J. Biomed. Mater. Res. B Appl. Biomater..

[32] Meningaud J.P., Spahn F., Donsimoni J.M. (2012). After Titanium, Peek?. Rev. Stomatol. Chir. Maxillofac..

[33] Godara A., Raabe D., Green S. (2007). The influence of sterilization processes on the micromechanical properties of carbon fiber-reinforced PEEK composites for bone implant applications. Acta Biomater..

[34] Mishra S., Chowdhary R. (2019). PEEK Materials as an Alternative to Titanium in Dental Implants: A Systematic Review. Clin. Implant. Dent. Relat. Res..

[35] Najeeb S., Bds Z.K., Bds S.Z., Bds M.S. (2013). Bioactivity Osseointegration of PEEK Are Inferior to Those of Titanium: A Systematic Review. J. Oral Implantol..

[36] Popa D., Constantiniuc M., Earar K., Mercut V., Scrieciu M., Buduru S., Luca E., Negucioiu M. (2019). Review of Different Materials that can be CAD/CAM Processed Description, chemical composition, indications in dentistry areas. Rev. Chim..

[37] Reyal S.S., Rajamani V.K., Gowda E.M., Shashidhar M.P. (2021). Comparative prospective clinical evaluation of computer aided design/computer aided manufacturing milled BioHPP PEEK inlays and Zirconia inlays. J. Indian. Prosthodont. Soc..

[38] Hallmann L., Mehl A., Sereno N., Hämmerle C.H. (2012). The improvement of adhesive properties of PEEK through different pre-treatments. Appl. Surf. Sci..

[39] Pacurar M., Bechir E.S., Suciu M., Bechir A., Biris C.I., Mola F.C., Gioga C., Dascalu I.T., Ormenisan A. (2016). The Benefits of Polyether-Ether-Ketone Polymers in Partial Edentulous Patients. Mater. Plast..

[40] Iyer R.S., Suchitra S.R., Coutinho A.C., Priya A. (2019). BioHPP: Properties and applications in prosthodontics a review. J. Res. Dent..

[41] Reda R., Zanza A., Galli M., De Biase A., Testarelli L., Di Nardo D. (2022). Applications and Clinical Behavior of BioHPP in Prosthetic Dentistry: A Short Review. J. Compos. Sci..

[42] Georgiev J., Vlahova A., Kissov H., Aleksandrov S., Kazakova R. (2018). Possible application of BioHPP in prosthetic dentistry: A literature review. J. IMAB.

[43] (2005). Rubber, Vulcanized or Thermoplastic—Determination of Tensile Stress-Strain Properties.

[44] (2005). Plastics and Ebonite—Determination of Indentation Hardness by Means of a Durometer (Shore Hardness).

[45] Baeana L.M., Zuleta E.C., Calderon J.A. (2018). Evaluation of the Stability of Polymeric Materials Exposed to Palm Biodiesel and Biodiesel–Organic Acid Blends. Polymers.

[46] Lim K., Hayat M.D., Jena K.D., Zhang W., Cao P. (2022). On amine treated polyoxymethylene (POM) blends with low formaldehyde emission for metal injection moulding (MIM). J. Mater. Sci..

[47] Kwon G., Kim H., Gupta KCh Kang I.-H. (2018). Enhanced Tissue Compatibility of Polyetheretherketone Disks by Dopamine-Mediated Protein Immobilization. Macromol. Res..

[48] Gaitanelis D., Worrall Ch Kazilas M. (2022). Detecting, characterising and assessing PEEK’s and CF-PEEK’s thermal degradation in rapid high-temperature processing. Polym. Degrad. Stab..

[49] Balestra D., Lowther M., Goracci C., Mandurino M., Cortili S., Paolone G., Louca C., Vichi A. (2024). 3D Printed Materials for Permanent Restorations in Indirect Restorative and Prosthetic Dentistry: A Critical Review of the Literature. Materials.

[50] Lin T.W., Corvelli A.A., Frondoza C.G., Roberts J.C., Hungerford D.S. (1997). Glass peek composite promotes proliferation and osteocalcin production of human osteoblastic cells. J. Biomed. Mater. Res..

[51] Steinberg E.L., Rath E., Shlaifer A., Chechik O., Maman E., Salai M. (2013). Carbon fiber reinforced PEEK Optima—A composite material biomechanical properties and wear/debris characteristics of CF-PEEK composites for orthopedic trauma implants. J. Mech. Behav. Biomed. Mater..

[52] Jockisch K.A., Brown S.A., Bauer T.W., Merritt K. (1992). Biological response to chopped-carbon-fiber-reinforced peek. J. Biomed. Mater. Res..

[53] Sandler J., Werner P., Shaffer M.S., Demchuk V., Altstädt V., Windle A.H. (2002). Carbon-nanofibre-reinforced poli(ether ether ketone) composites. Compos. Part. A Appl. Sci. Manuf..

[54] Schwitalla A.D., Spintig T., Kallage I., Müller W.D. (2015). Flexural behavior of PEEK materials for dental application. Dent. Mater..

