# Supplementary material for: Comparison of the Performance Parameters of BioHPP® and Biocetal® Used in the Production of Prosthetic Restorations in Dentistry—Part I: Mechanical Tests: An In Vitro Study
Source: Materials (Basel). 2025 Jan 26;18(3):561. doi: 10.3390/ma18030561 (PMC11818239; doi:10.3390/ma18030561)
Supplement: Supplementary file 1 [file materials-18-00561-s001.zip › materials-3384203-supplementary.pdf]

Supplementary Information

Comparison of the Performance Parameters of BioHPP and Biocetal Used in the Production of Prosthetic Restorations in Dentistry. Part I: Mechanical Tests: An In Vitro Study

Robert Kowalski [1], Wojciech Frąckiewicz [2,*], Magdalena Kwiatkowska [3], Małgorzata Światłowska-Bajzert [1] and Ewa Sobolewska [1]

[1]Department of Dental Prosthetics, Faculty of Medicine and Dentistry, Pomeranian Medical University in Szczecin, Av. Powstańców Wlkp. 72, 70-111 Szczecin, Poland;

[2]Ra-Dent Stomatologia Protetyka, Bolesława Krzywoustego Street 19/5, 70-252 Szczecin, Poland;

[3]Faculty of Mechanical Engineering and Mechatronics, West Pomeranian University of Technology in Szczecin, Av. Piastów 19, 70-310 Szczecin, Poland.

*Correspondence: woj.frackiewicz@gmail.com

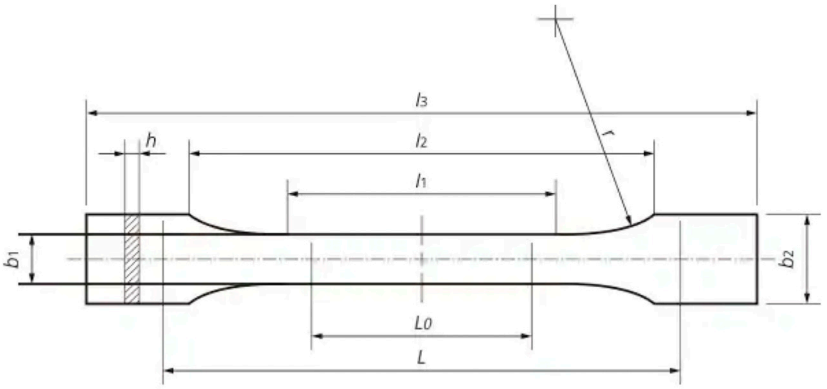

Figure S1. Sample design.

Table S1. Injection molding parameters

Material	Temperature [°C]				Pressure [MPa]		Total injection time [s]
	Feed hopper	Heating cylinder	Nozzle	Mold	Injection	Clamping	
**Biocetal**	180	200	210	40	90	35	42
**BioHPP**	300	390	400	120	150	100	56

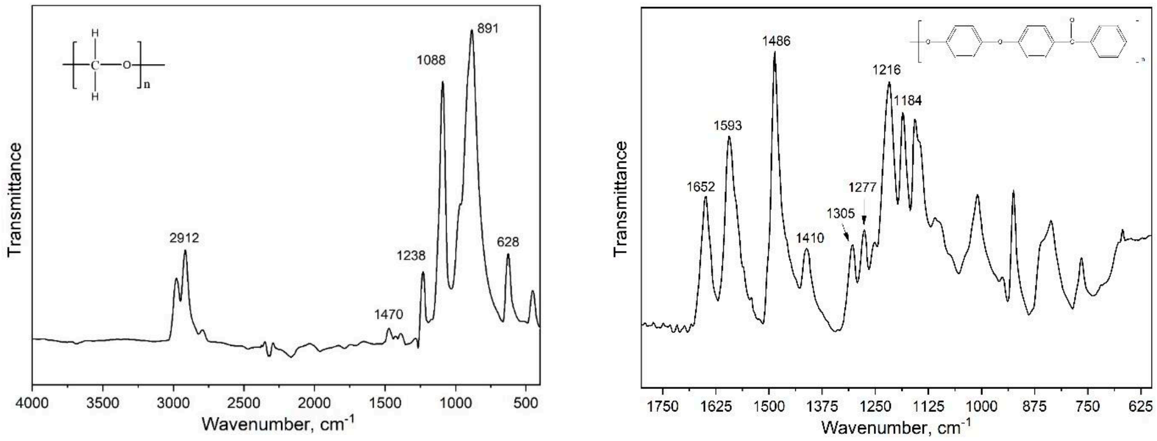

(**a**) (**b**)

Figure S2. ATR-FTIR spectra of Biocetal (a) and BioHPP (b)

Table S2. Statistical analysis of hardness determination results:

**Hardness marking**										
**Material**	n	min	max	median	q1	q3	mean	sd	se	statistic
**Biocetal**	10	76,00	79,00	78,00	77,00	79,00	77,90	1,10	0,35	t=-6,22
**BioHPP**	10	80,00	85,00	82,50	80,20	84,00	82,30	1,95	0,62	p<0,0001

Table S3. Statistical analysis of the results of tensile tests.

**Tensile test - elongation at yield point**										
**Material**	n	min	max	median	q1	q3	mean	sd	se	statistic
**Biocetal**	7	11.30	12.30	11.70	11.60	11.80	11.70	0.30	0.11	t=63.52
**BioHPP**	8	3.84	4.17	4.08	3.96	4.13	4.04	0.13	0.05	p<0.0001
**Tensile test - stress at yield point**										
**Material**	n	min	max	median	q1	q3	mean	sd	se	statistic
**Biocetal**	7	66.30	67.40	67.00	66.80	67.20	67.00	0.38	0.14	t=-26.3
**BioHPP**	8	81.40	87.70	84.60	84.30	85.50	84.80	1.87	0.66	p<0.0001
**Tensile test - stress at break**										

Material	n	min	max	median	q1	q3	mean	sd	se	statistic
**Biocetal**	7	53.00	62.00	59.00	56.00	59.00	57.70	2.98	1.13	t=-1.85
**BioHPP**	7	43.00	85.00	76.00	59.50	80.50	69.10	16.00	6.06	p=0.11
**Tensile test - relative elongation at break**										
**Material**	n	min	max	median	q1	q3	mean	sd	se	statistic
**Biocetal**	7	29.30	67.00	40.00	35.80	55.10	45.40	14.10	5.34	t=-7.00
**BioHPP**	7	76.30	96.40	87.20	83.70	91.80	87.30	7.09	2.68	p<0.0001
**Tensile test - Elastic modulus**										
**Material**	n	min	max	median	q1	q3	mean	sd	se	statistic
**Biocetal**	7	2.89	3.36	3.10	3.09	3.12	3.11	0.14	0.05	t=-16.70
**BioHPP**	7	4.93	5.84	5.35	5.13	5.48	5.34	0.32	0.12	p<0.0001

Table S4. Statistical analysis of the results of determining the maximum stress and relative strain in the bending test.

**Flexural strength test - maximum stress**										
**Material**	n	min	max	median	q1	q3	mean	sd	se	statistic
**Biocetal**	3	92.70	93.20	93.20	92.90	93.20	93.00	0.30	0.17	t=41.4
**BioHPP**	4	134.00	139.00	137.00	136.00	138.00	137.00	2.09	1.04	p<0.0001
**Flexural strength test - relative deformation at maximum stress**										
**Material**	n	min	max	median	q1	q3	mean	sd	se	statistic
**Biocetal**	3	7.62	7.89	7.68	7.65	7.79	7.73	0.14	0.08	t=11.4
**BioHPP**	5	5.96	6.51	6.28	6.24	6.45	6.29	0.22	0.09	p<0.0001

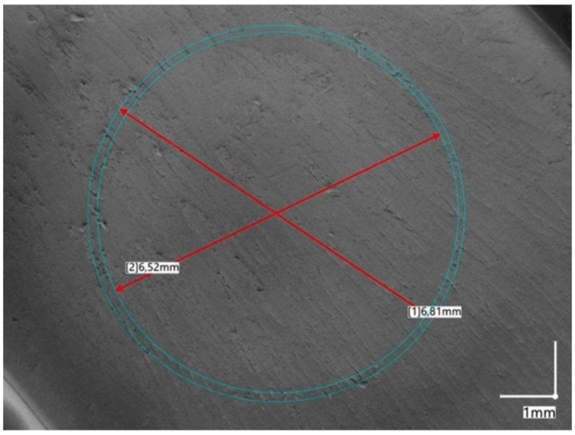

(**a**)

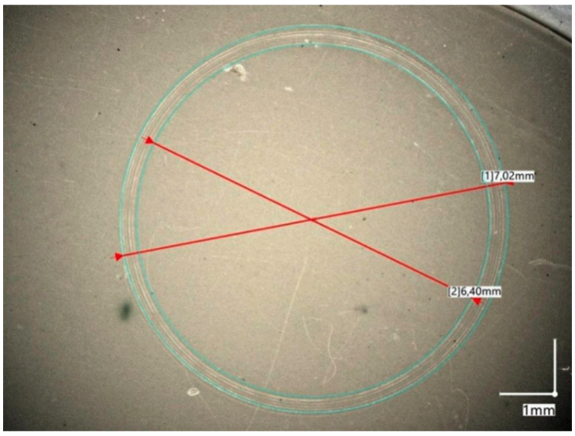

(**b**)

Figure S3. A microscopic image of the wear trace of a representative sample of BioHPP **(a)** and Biocetal **(b)**, the diameter of which was used to calculate the wear rate (K) in the "wet" test.
